# Lectin from *Crataeva tapia* Bark Improves Tissue Damages and Plasma Hyperglycemia in Alloxan-Induced Diabetic Mice

**DOI:** 10.1155/2013/869305

**Published:** 2013-11-13

**Authors:** Amanda Alves da Rocha, Tiago Ferreira da Silva Araújo, Caíque Silveira Martins da Fonseca, Diógenes Luís da Mota, Paloma Lys de Medeiros, Patrícia Maria Guedes Paiva, Luana Cassandra Breitenbach Barroso Coelho, Maria Tereza dos Santos Correia, Vera Lúcia de Menezes Lima

**Affiliations:** ^1^Departamento de Bioquímica, Centro de Ciências Biológicas, Universidade Federal de Pernambuco, Avenida Professor Moraes Rego S/N, 50670-420 Recife, PE, Brazil; ^2^Departamento de Histologia e Embriologia, Centro de Ciências Biológicas, Universidade Federal de Pernambuco, Avenida Professor Moraes Rego S/N, 50670-420 Recife, PE, Brazil

## Abstract

*Crataeva tapia* is a plant popularly used for diabetes treatment, in Brazil. Progressive decline in renal and hepatic functions has been described in patients with diabetes mellitus, and mortality rate is increased in patients with chronic liver and renal disease. This study aimed to evaluate whether *Crataeva tapia* bark lectin (CrataBL) improves hyperglycemia and renal and hepatic damage in diabetic mice. CrataBL was purified by ion exchange chromatography on CM-cellulose, and intraperitoneal administration of CrataBL to alloxan-induced diabetic mice at dose of 10 mg/Kg/day and 20 mg/Kg/day for 10 days significantly reduced serum glucose levels by 14.9% and 55.9%, respectively. Serum urea, creatinine, aspartate aminotransferase, and alanine aminotransferase were also significantly reduced after treatment with both doses of CrataBL. Furthermore, histological analysis of liver, kidney, and pancreas revealed an improvement in the tissue morphology upon treatment with CrataBL. The results suggest that CrataBL has a beneficial hypoglycemic activity and improves the renal and hepatic complications of diabetes. Therefore, this lectin may be a promising agent for the treatment of diabetes, and this might be the basis for its use in the folk medicine as an alternative treatment to manage diabetes-related complications such as hyperglycemia and tissue damage.

## 1. Introduction

*Crataeva tapia *(also known as* Crateva tapia*), a plant ofCapparidaceae family, is commonly found in Pluvial Tropical Atlantic Forest and Pantanal Tropical Forest in Brazil [1]. *C. tapia* is known by Northeast Brazilian people as “paudalho” or “tapiá” and its bark is largely used in the folk medicine for the treatment of diabetes. Recently, a lectin with a molecular weight of 40 kDa (CrataBL) was purified from the aqueous extract of *Crataeva tapia* bark [2]. Lectins are carbohydrate binding proteins, of nonimmunogenic origin, that bind specifically and reversibly to different types of carbohydrates or glycoproteins and can be obtained from several sources, mainly from vegetal [3]. Several plant lectins have been demonstrated to possess a variety of biological activities including antitumor [4–6], anti-inflammatory [7, 8], antimicrobial [9–11], analgesic [4], antioxidant [3] insecticidal [2, 12–14], anticoagulant [15], and hypoglycemic [16, 17]. 

Diabetes mellitus is a chronic disease considered to be one of the five leading causes of death in the world, and it is a complex metabolic disease with great development of pathological changes in many tissues [18]. The disease is characterized by alteration in the carbohydrate metabolism resulting in an increase of the glucose levels [19]. Approximately 360 million of adult people have diabetes, corresponding to 8.3% of the world with diabetes, and this is projected to rise to 552 million by 2030, corresponding to 9.9% of the world population [20]. The hyperglycemia in diabetes produces superoxide anions, which generate hydroxyl radicals, promoting cell membrane damages as a result of lipid peroxidation and protein glycation of membrane [18]. In diabetic individuals the major alterations occur in renal and hepatic tissue and have been associated with functional and morphological damage in these organs [21, 22]. Among the common complications of diabetes the nephropathy is a chronic disease that affects 40% of individuals. Diabetic nephropathy is responsible for 50% of chronic renal failure cases [23]. Furthermore, hepatic dysfunction promoted by diabetes can result in nonalcoholic steatosis, hepatomegaly amongst others [24].

Studies have reported that the doubts about the efficacy and safety of some of the oral hypoglycemic agents have prompted a search for safer and more effective drugs in the treatment of diabetes [25]. Thus, the aim of the present study was to investigate whether CrataBL from *C. tapia* bark is a metabolite with potential antihyperglycemic activity.

## 2. Material and Methods

### 2.1. Chemicals

Alloxan monohydrated and CM-cellulose was purchased from Sigma-Aldrich Chemical Company, St. Louis, MO, USA. Insulin (Humolin N) was purchased from Lilly, Brazil. All the other chemicals used were in an analytical grade.

### 2.2. Plant Material

*C. tapia *barks were collected from the Recife City, PE, Northeast Brazil. The plant was identified by *Instituto Agronômico de Pernambuco* (IPA) and a voucher specimen was deposited (n° 61.415).

### 2.3. Purification of Crataeva Tapia Bark Lectin

*C. tapia *bark lectin was obtained through a sequential purification protocol as previously reported by Araújo et al. [2]. Powdered bark (10 g) was suspended in 0.15 M NaCl (100 mL). After homogenization in a magnetic stirrer (16 h at 4°C), followed by filtration through gauze and centrifugation (4,000 ×g, 15 min), the supernatant (crude extract) was taken as starting material. Soluble proteins in crude extract were fractionated with ammonium sulphate and the 30–60% precipitate fraction (30–60 F) was submitted to dialysis (3,500 Da cut-off membrane, 4°C) against distilled water (2 h) followed by 10 mM citrate-phosphate buffer pH 5.5 (2 h). The 30–60 F was applied (11 mg of protein, hemagglutinating activity of 1024) into a CM-cellulose chromatography column (5.2 cm × 1.6 cm) equilibrated with 10 mM citrate-phosphate buffer pH 5.5 at flow rate of 20 mLh^−1^. The unabsorbed proteins were eluted with the buffer solution until the absorbance at 280 nm was lower than 0.05; CrataBL was eluted with 0.5 M NaCl. Protein concentration was determined according to Lowry et al. [26] using bovine serum albumin as standard.

### 2.4. Animals

Female albino Swiss mice (*Mus musculus*), six weeks of age, weighing 30 ± 5 g, bred in the Biotherium of *Departamento de Antibióticos*, UFPE, Brazil, were housed in colony cages (six mice per cage) at room temperature of 22 ± 2°C, relative humidity 40–60%, and 12 h light and 12 h dark cycle. The mice were fed standard rodent diet (Labina, Purina Brazil Ltd., Brazil) and water *ad libitum*. The experimental protocol was approved by the Animal Care and Use Committee at the Federal University of Pernambuco, Brazil (CEEA-UFPE-Ofício n° 40/06). All experimental procedures were conducted in accordance with the ethical guidelines for Care and Use of Laboratory Animals. 

### 2.5. Induction of Diabetes in Mice

Experimental diabetes was induced in overnight-fasted mice by a single intraperitoneal injection of freshly prepared alloxan monohydrated (80 mg/kg in 0.9% NaCl solution). After alloxan administration, all animals were relocated to their cages and given free access to food and water. Diabetes was confirmed by measuring the fasting blood glucose levels 72 h after alloxan injection. The mice with serum glucose of >250 mg/dL were considered diabetic and were included in the study.

### 2.6. Experimental Design

The mice were split into four groups (*n* = 6, for group) as follows:

 Group (I)—normoglicemic mice receiving saline solution (0.9%), as control group;
 Group (II)—diabetic control mice, named diabetic nontreated;
 Group (III)—diabetic mice treated with CrataBL (10 mg/kg/day, intraperitoneally) in saline solution (0.9%) for 10 days, named diabetic treated 10;
 Group (IV)—diabetic mice treated with CrataBL (20 mg/kg/day, intraperitoneally) in saline solution (0.9%) for 10 days, named diabetic treated 20;
 Group (V)—diabetic mice treated with insulin (10 mg/kg/day, intraperitoneally) for 10 days, named diabetic insulin treated.

Before and at the end of the experimental period, overnight fasting mice were anaesthetized with 2% xylazine hydrochloride (10 mg/kg) and 10% ketamine hydrochloride (115 mg/kg); blood samples were withdrawn with a capillary from mice-cavernous sinus for biochemical parameters determination [27]. The mice were sacrificed by cervical dislocation. Thereafter, pancreas, liver, and kidneys were excised and immediately fixed in 10% neutral buffered formalin for histological analysis.

### 2.7. Effect of CrataBL on Biochemical Data

Mice blood samples were centrifuged at 2,500 g for 15 min at 4°C (Sorvall RC6, NC, US). Sera were obtained and the levels of the glucose, urea, creatinine, aspartate aminotransferase (AST), and alanine aminotransferase (ALT) were measured by enzymatic colorimetric methods (Labtest Diagnostica, Brazil/SA) in a chemistry autoanalyzer (COBAS 6000, Roche Diagnostics, England).

### 2.8. Histological Analysis of Pancreas, Kidneys, and Liver

Pancreas, kidney, and liver from all groups were subjected to standard routine tissue processing technique: dehydrated in gradual ethanol (50–100%), cleared in xylene, and embedded in paraffin. Sections of 5 **µ**m thickness were cut from each block and stained with haematoxylin-eosin for histological examination. Prepared slides were studied by light microscopy and all sections were evaluated for the tissue injury.

### 2.9. Statistical Analysis

Values were expressed as the mean ± SD. Multiple comparisons were analyzed by one-way ANOVA followed by Tukey's *post hoc* test. For all analysis the 0.05 level of probability was used as the criterion of significance. The analyses were carried out using software PRISMA (GraphPad Software, Inc., San Diego, CA, version 5.01). 

## 3. Results and Discussion

### 3.1. Effect of CrataBL on Fasting Glucose

Diabetes is a complex metabolic disorder with a characteristic modulation of glucose metabolism. Chronic hyperglycemia promotes tissue damage which can be found in many organs and systems, with consequent often serious disease [28]. Alloxan, a prominent diabetogenic chemical with ability to generate reactive oxygen species formation that induce death of *β* cell of the pancreas by necrosis [29], is considered a good model for reproducible induction of the metabolic state of this disease in experimental animals [30–33]. Thus, in this study the mice subjected to alloxan injection showed symptoms of severe diabetes such as hyperglycemia. Insulin treatment, as a positive control, validates our model by showing the improvement in diabetes. 

In a previous study, the acute toxicity of CrataBL was determined in mice; at the doses from 300 mg/kg to 2,000 mg/kg, mice did not present weight loss or death, and LD_50_ of CrataBL was 2,500 mg/kg [4]. Therefore, CrataBL concentrations used in the present study are considered safe, without problem of toxicity, and indicate that the lectin is a potential pharmaceutical substance. 

**Figure 1:**
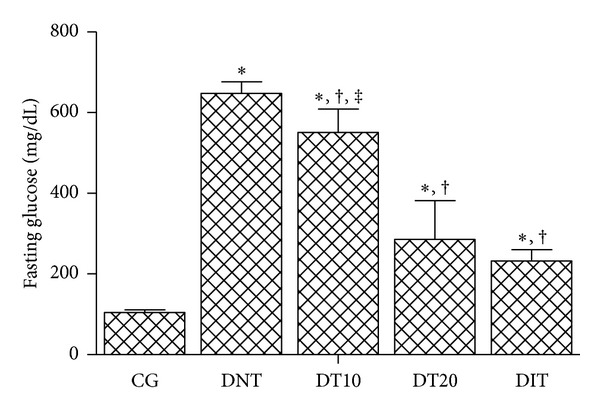
Fasting serum glucose levels in diabetic mice after treatment with CrataBL. CG: control group; DNT: diabetic nontreated; DT10: diabetic treated with CrataBL (10 mg/kg); DT20: diabetic treated with CrataBL (20 mg/kg); DIT: diabetic treated with insulin (10 mg/kg). *P < 0.05 versus CG; ^†^ *P* < 0.05 versus DNT; ^‡^ *P* < 0.05 versus DIT.

As demonstrated in Figure 1, CrataBL proved to be an effective hypoglycemic agent after 10 days of treatment and showed significant antihyperglycemic activity in a dose-dependent manner, in alloxan-induced diabetic mice, and at the dose of 20 mg/kg/day it exhibited better glucose reduction (56%) than 10 mg/kg/day (15%), and it was similar to that found by the treatment with the standard drug, insulin (64%), without no significant difference (*P* > 0.05). Studies with soya bean lectin reported a decrease of 17.3% in blood glucose, and it was suggested that this effect is due to an increase in pancreatic growth stimulated by the lectin [34]. Wang et al. [35] demonstrated that *Agaricus bisporus* lectin administration could partially reverse the impaired *β*-cell growth potential by regulating cell cycle proteins (cyclin D1, cyclin D2, and Cdk4). So, induction of pancreatic *β*-cell proliferation by lectins suggests the therapeutic potential in decreasing blood glucose and treating experimental diabetes mellitus [34, 35]. 

Medicinal plants are gaining wide acceptably worldwide because they are the potential sources of bioactive agents in use as pharmaceutics. In a fast changing world, a number of procedures to evaluate hypoglycemia as well as the kidney and liver damage have been used to investigate the effectivity of new natural agents which are explored by experts and clinicians [36–39].

### 3.2. Effects of CrataBL on Markers of Kidney Damage

As shown in Table 1, levels of urea and creatinine known as kidney function markers were significantly increased in sera of alloxan-induced diabetic mice, in comparison with normal mice. After 10 days of treatment with CrataBL, the levels of urea and creatinine significantly decreased. The diabetic mice treated with CrataBL at doses of 10 and 20 mg/kg reduced serum levels of urea by 20.7% and 25.3%, respectively, and the same doses decreased creatinine concentration by 15.4% and 17.9%, respectively. Insulin, the positive control for treatment, decreased these markers of renal damage by 26.8% and 17.9%. Our results are in agreement with recent reports by Kumar et al. [39], Omara et al. [40], and Yankuzo et al. [41] who demonstrated that renal damage can be ameliorated when the levels of serum urea and creatinine are decreased by treatment with extracts of medicinal plants. 

Kidney damage is usually associated with diabetes. In the initial course of disease the presence of hypertrophy of the glomeruli and tubular cells, matrix expansion, and enhanced renal blood flow is common, and these alterations have been postulated to cause loss of renal function [40, 41]. High levels of urea and creatinine are usually reported as one of the most sensitive markers of kidney damage, and it is reported that renal hypertrophy in diabetic animals is caused by an increased formation of advanced glycation end products and accumulation of glycogen granules in distal tubules [42, 43]. 

Thus, our results clearly indicate that CrataBL possesses an effective potential to improve kidney damage induced by alloxan-diabetes.

### 3.3. Effects of CrataBL on Markers of Liver Damage

**Figure 2:**
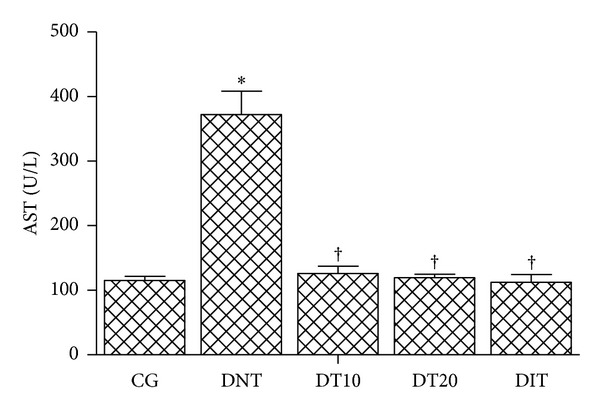
Serum aspartate aminotransferase levels in diabetic mice after treatment with CrataBL. CG: control group; DNT: diabetic nontreated; DT10: diabetic treated with CrataBL (10 mg/kg); DT20: diabetic treated with CrataBL (20 mg/kg); DIT: diabetic treated with insulin (10 mg/kg). *P < 0.05 versus CG; ^†^ *P* < 0.05 versus DNT.

**Figure 3:**
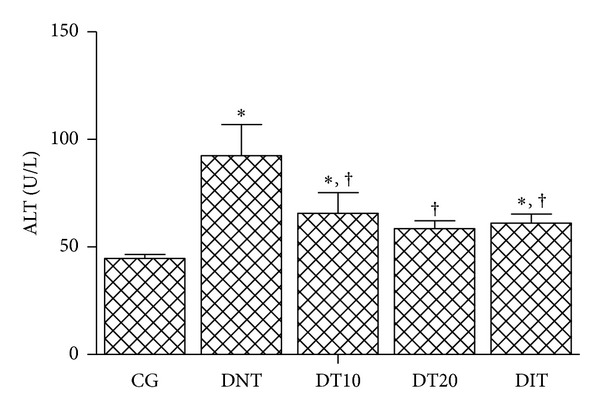
Serum alanine aminotransferase levels in diabetic mice after treatment with CrataBL. CG: control group; DNT: diabetic nontreated; DT10: diabetic treated with CrataBL (10 mg/kg); DT20: diabetic treated with CrataBL (20 mg/kg); DIT: diabetic treated with insulin (10 mg/kg). *P < 0.05 versus CG; ^†^ *P* < 0.05 versus DNT.

As compared to the control groups, the activities of the markers of liver damage serum AST and ALT were significantly (*P* < 0.05) reduced in alloxan-induced diabetic mice after treatment with 10 or 20 mg/kg of CrataBL; the activity of AST was reduced by 66.2% and 67.9%, respectively (Figure 2) and ALT activity was decreased by 28.9% and 36.6%, respectively (Figure 3). These percentages of reduction were similar to those observed with insulin treatment. Therefore, administration of CrataBL for 10 days reversed the elevated levels of liver marker enzymes, which reflects the capability to conserve the membrane integrity of cellular and mitochondrial membranes of hepatocyte in alloxan-diabetic mice treated with this lectin. 

Our results are in agreement with those of Mansour et al. [25] who reported that hepatic damage can be improved by decreasing the levels of serum AST and ALT in alloxan-induced diabetic rats subjected to treatment with herbal bioactive agents. It is well known that liver is the focal organ of oxidative and detoxifying processes [22]. Liver diseases are a high problem of health worldwide and the release of intracellular localized marker enzymes such as AST and ALT into the blood when cell and mitochondria are subjected to injury indicates hepatocytes damage [44, 45]. Furthermore, the elevated serum levels of AST and ALT in nontreated diabetic mice (Figures 2 and 3) indicate that alloxan caused liver damage and loss of the functional integrity of the hepatocyte membranes, as also evidenced in a study reported by Rajesh and Latha [45] about hepatotoxicity of polyherbal formulation.

As indicated by serum levels of AST and ALT CrataBL is able to improve liver damage. 

### 3.4. Effects of CrataBL on the Histopathological Changes of the Pancreas, Liver, and Kidneys

**Figure 4:**
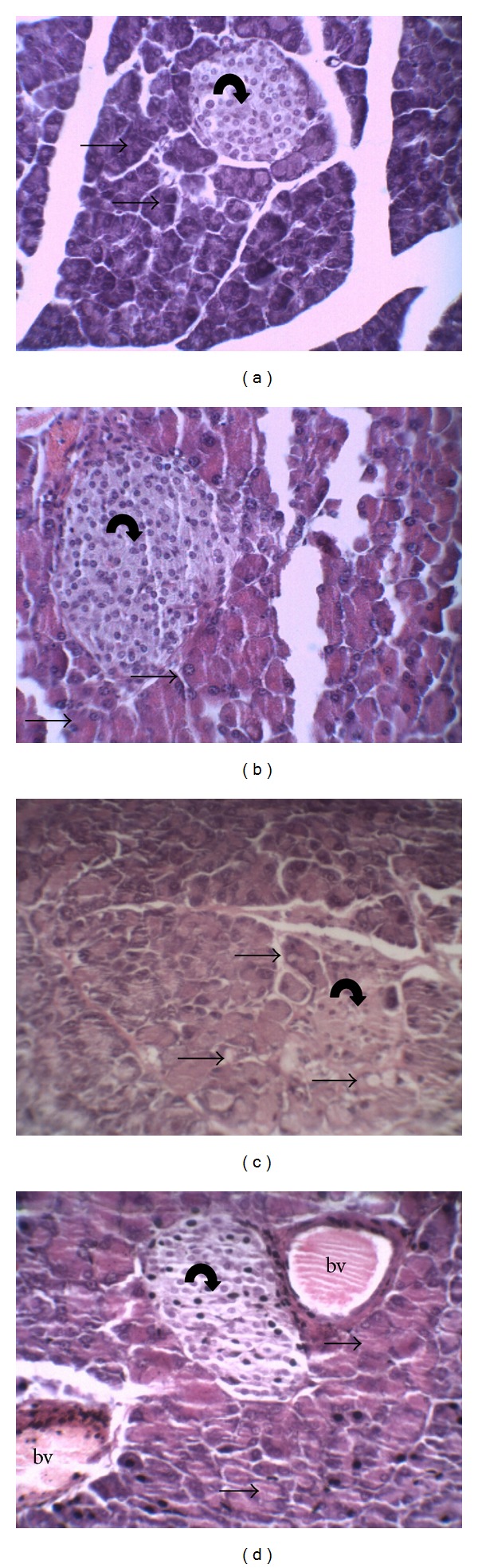
Histopathological changes in pancreatic tissue. (a) Control group—preserved pancreatic islet of Langerhans (curved arrow) and preserved pancreatic acinar cells (straight arrows); (b) diabetic nontreated—atrophic pancreatic islet of Langerhans with few cells (curved arrow) and the presence of some vacuoles in the pancreatic acinar cells (straight arrows); (c) diabetic treated with CrataBL (10 mg/kg)—pancreatic islet of Langerhans (curved arrow) and preserved pancreatic acinar cells (straight arrows); (d) diabetic treated with CrataBL (20 mg/kg)—preserved pancreatic islet of Langerhans (curved arrow) and preserved pancreatic acinar cells (straight arrows) and blood vessel (bd). Haematoxylin-eosin: 400x.

The structure of the pancreas of the control and diabetic mice are shown in Figure 4. Pancreas of control group showed normal pancreatic islet of Langerhans and acinar cells (Figure 4(a)). By contrast, in alloxan-induced diabetic mice the acinar cells were altered with presence of vacuoles; furthermore deterioration of pancreatic islets was also observed (Figure 4(b)). CrataBL (10 mg/kg) treatment increased the number of pancreatic islets as compared to that of diabetic nontreated animals (Figure 4(c)). Interestingly, pancreatic section of diabetic mice treated with CrataBL (20 mg/kg) showed pancreatic islet similar to that of the control group (Figure 4(d)).

The histopathological analysis of pancreas isolated from mice administrated with alloxan alone revealed tissue damage with deterioration of pancreatic islets. In this connection, it may be observed that several authors reported such changes in pancreas tissues of mice exposed to prominent diabetogenic alloxan for its ability to induce reactive oxygen species (ROS) formation, resulting in the selective necrosis of beta cells in pancreatic islets [29, 39, 46, 47]. However, the diabetic animals treated with lectin from *C. tapia* bark showed normal architecture of pancreatic tissue, suggesting the regeneration of pancreatic islet by CrataBL administrations. The ability of lectins to stimulate pancreatic growth has been reported [48]. The regenerative action of CrataBL corroborates with *Agaricus bisporus* lectins (ABL). The ABL administration could partially reverse the impaired *β*-cell growth potential by induction of pancreatic islet proliferation [35]. Thus, the antidiabetic effect observed by CrataBL administration suggests the therapeutic potential in preventing and/or treating diabetes.

**Figure 5:**
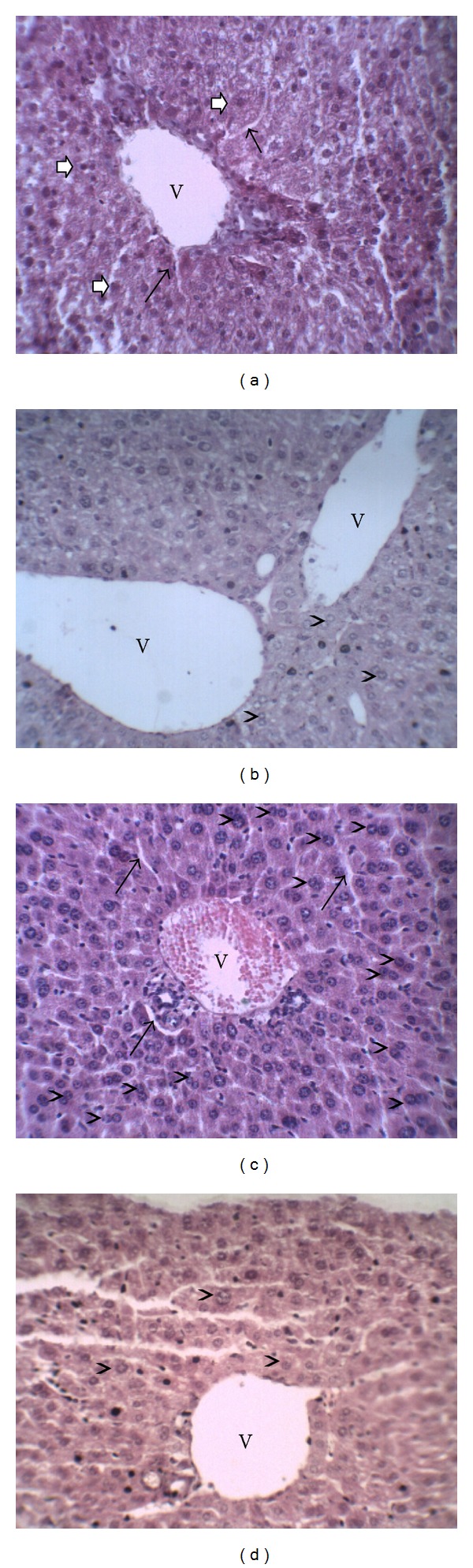
Histopathological changes in hepatic tissue. (a) Control group—centrilobular vein (V), preserved hepatocytes (white arrows), and sinusoidal capillaries (thin arrows); (b) diabetic nontreated—centrilobular vein with many red blood cells (V), intense mitotic activity in hepatocytes (arrowheads), and the presence of sinusoidal capillaries (thin arrows); (c) diabetic treated with CrataBL (10 mg/kg)—centrilobular vein (V) and considerable mitotic activity in hepatocytes (arrowheads); (d) diabetic treated with CrataBL (20 mg/kg)—centrilobular vein (V) and preserved hepatocytes (arrowheads). Haematoxylin-eosin: 400x.

Figure 5 shows the photomicrographs of hepatic tissues of control group and diabetic experimental groups. The section of liver tissue of control mice demonstrates preserved hepatocytes, centrilobular vein, and sinusoidal capillaries (Figure 5(a)). In the alloxan-induced diabetic mice the histopathological analysis of hepatic tissue shows intense mitotic activity in hepatocytes (Figure 5(b)). CrataBL (10 mg/kg) treatment exhibited considerable mitotic activity in hepatocytes (Figure 5(c)). Similar to the control group, diabetic mice treated with CrataBL (20 mg/kg) also revealed an equivalent architecture of hepatic tissue (Figure 5(d)).

**Figure 6:**
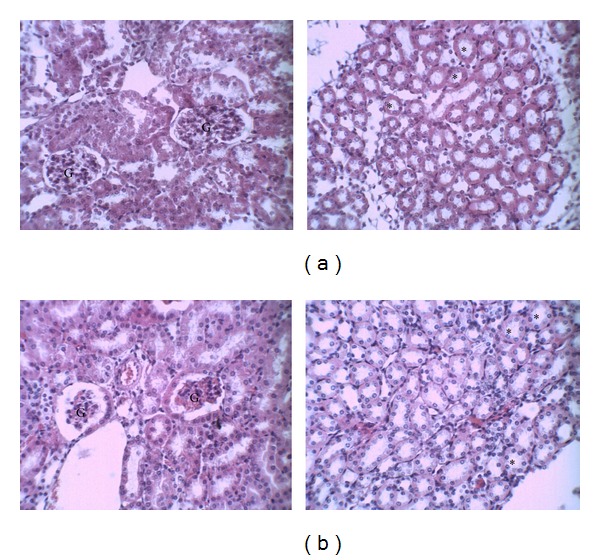
Histopathological changes in renal tissue of the normal and diabetic mice. (a) Control group—renal glomeruli (G) with preserved subcapsular spaces (left) and collecting tubules (stars) without changes in the medullary region (right); (b) diabetic nontreated—retracted glomerular tufts (G) with increased subcapsular space and evident thickening of parietal layer of Bowman's capsule due to have been entirely replaced by the cuboidal cells (left) and preserved collecting tubules (stars) (right). Haematoxylin-eosin: 400x.

**Figure 7:**
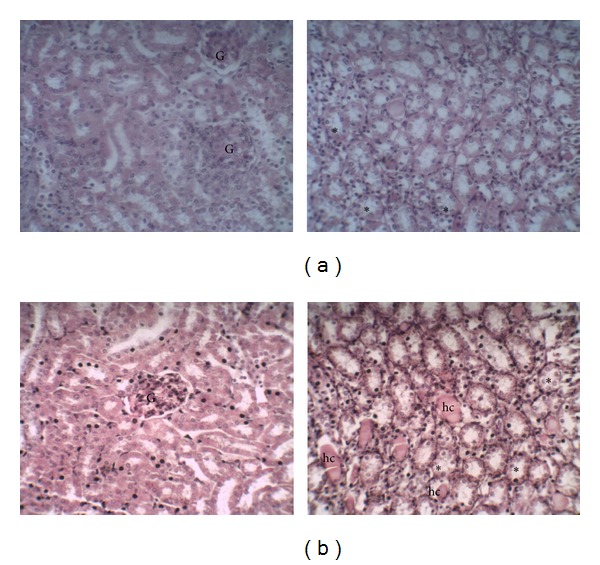
Histopathological changes in renal tissue of the diabetic mice treated with CrataBL. (a) Diabetic treated with CrataBL (10 mg/kg)—renal glomeruli (G) with irregular subcapsular spaces and some distinctly collapsed (left) and collecting tubules with slight swelling of the tubular epithelium (stars) (right); (b) diabetic treated with CrataBL (20 mg/kg)—preserved renal glomeruli (G) (left) and presence in the medullary region of collecting tubules (stars) with evident swelling of the tubular epithelium and hyaline casts (hc) (right). Haematoxylin-eosin: 400x.

The photomicrographs of renal tissues are represented in Figures 6 and 7. Figures 6(a) and 6(b) represent the renal tissues of control group and diabetic nontreated group, respectively. Kidneys of control group show normal architecture of tissue with preserved subcapsular spaces in glomeruli and collecting tubules without change in the medullary region. Differently, the renal tissue of alloxan-induced diabetic mice shows retracted glomerular tufts with increased subcapsular space and evident thickening of Bowman's membrane due to the cuboid appearance of epithelial cells. In kidneys of alloxan-induced mice treated with CrataBL (10 mg/kg) renal glomeruli were evident with irregular subcapsular spaces and some distinctly collapsed (Figure 7(a)). However, renal sections of diabetic mice treated with CrataBL (20 mg/kg) show preserved renal glomeruli and presence in the medullary region of collecting tubules with evident swelling of the tubular epithelium and hyaline casts presence (Figure 7(b)).

The elevated levels of glucose contribute to the generation of ROS in the diabetes, which promotes to the increase of oxidative stress in various organs and tissues [49, 50]. In addition, the hyperglycemia provokes hepatic and renal damage and consequently has been associated with histological and functional alterations and liver and kidneys [51, 52]. In fact, these organs are the focal of important organic functions and damage promoted by diabetes can result in severe complications with nephropathy and nonalcoholic steatosis [23, 24]. The current study demonstrated that CrataBL treatment improves the hepatic and renal histologic damage induced by diabetes. These findings correlated with improved biochemical markers of liver and renal functions by CrataBL. Taken together, these results may contribute to a better understanding of the regenerative effect of CrataBL in pancreas and protective in liver and kidneys, emphasizing the utilization of this lectin in the treatment of complications associated with diabetes mellitus. 

## 4. Conclusion

Our results indicate that CrataBL is a good agent in controlling diabetes induced by alloxan and improves the damage on kidneys and liver tissues. The findings of this study also indicate that the active principle present in *C. tapia* is CrataBL, which is a lectin responsible for the antihyperglycemic activity found in this study and that could explain the basis for its use in the folk medicine as an alternative treatment for diabetes. Therefore, we conclude that CrataBL serves as an excellent candidate for an alternative therapy in the treatment of diabetes mellitus since it revealed an antidiabetic activity and other beneficial effects that ameliorate diabetes and associated complications.

## Figures and Tables

**Table 1:** Serum urea and creatinine levels in diabetic mice after treatment with CrataBL.

Groups	Urea	Creatinine
CG	34.3 ± 6.8	0.30 ± 0.01
DNT	58.9 ± 5.8*	0.39 ± 0.04*
DT10	46.7 ± 6.6^∗,†^	0.33 ± 0.05^†^
DT20	44.0 ± 2.9^∗,†^	0.32 ± 0.04^†^
DIT	43.1 ± 2.6^†^	0.32 ± 0.02^†^

CG: control group; DNT: diabetic nontreated; DT10: diabetic treated with CrataBL (10 mg/kg); DT20: diabetic treated with CrataBL (20 mg/kg); DIT: diabetic treated with insulin (10 mg/kg). *P < 0.05 versus CG; ^†^ *P* < 0.05 versus DNT.
